# The prevention of 2,4-dinitrochlorobenzene-induced inflammation in atopic dermatitis-like skin lesions in BALB/c mice by Jawoongo

**DOI:** 10.1186/s12906-018-2280-z

**Published:** 2018-07-13

**Authors:** Jin Mo Ku, Se Hyang Hong, Soon Re Kim, Han-Seok Choi, Hyo In Kim, Dong Uk Kim, So Mi Oh, Hye Sook Seo, Tai Young Kim, Yong Cheol Shin, Chunhoo Cheon, Seong-Gyu Ko

**Affiliations:** 1 Department of Science in Korean Medicine, Graduate School, Kyung Hee University, Kyungheedae-ro 26, Dongdaemun-gu, Seoul, 02447 Republic of Korea; 2 Department of Preventive Medicine, College of Korean Medicine, Kyung Hee University, Kyungheedae-ro 26, Dongdaemun-gu, Seoul, 02447 South Korea

**Keywords:** Atopic dermatitis, Jawoongo, 2,4-dinitrochlorobenzene, Cytokine, Inflammation

## Abstract

**Background:**

Jawoongo is an herbal mixture used in traditional medicine to treat skin diseases. This study aimed to investigate whether Jawoongo ameliorates Atopic dermatitis (AD)-like pathology in mice and to understand its underlying cellular mechanisms.

**Methods:**

AD was induced by 2, 4-Dinitrocholrlbenzene (DNCB) in BALB/c mice. Treatment with Jawoongo was assessed to study the effect of Jawoongo on AD in mice. Histological Analysis, blood analysis, RT-PCR, western blot analysis, ELISA assay and cell viability assay were performed to verify the inhibitory effect of Jawoongo on AD in mice.

**Results:**

We found that application of Jawoongo in an ointment form on AD-like skin lesions on DNCB-exposed BALB/c mice reduced skin thickness and ameliorated skin infiltration with inflammatory cells, mast cells and CD4+ cells. The ointment also reduced the mRNA levels of IL-2, IL-4, IL-13 and TNF-α in the sensitized skin. Leukocyte counts and the levels of IgE, IL-6, IL-10 and IL-12 were decreased in the blood of the DNCB-treated mice. Furthermore, studies on cultured cells demonstrated that Jawoongo exhibits anti-inflammatory activities, including the suppression of proinflammatory cytokine expression, nitric oxide (NO) production, and inflammation-associated molecule levels in numerous types of agonist-stimulated innate immune cell, including human mast cells (HMC-1), murine macrophage RAW264.7 cells, and splenocytes isolated from mice.

**Conclusion:**

These findings indicate that Jawoongo alleviates DNCB-induced AD-like symptoms via the modulation of several inflammatory responses, indicating that Jawoongo might be a useful drug for the treatment of AD.

**Electronic supplementary material:**

The online version of this article (10.1186/s12906-018-2280-z) contains supplementary material, which is available to authorized users.

## Background

Atopic dermatitis (AD) is the most common chronic inflammatory and chronically relapsing skin disease. The prevalence of AD has increased continuously, and approximately 10 million people worldwide are currently affected. The disease leads to a significantly reduced quality of life [1, 2]. The pathogenesis of AD is not well understood but appears to be associated with the activation of innate immune responses, including inflammation.

Common features of AD include excessive infiltration of inflammatory cells and granulated mast cells into AD skin lesions and high immunoglobulin E (IgE) levels and leukocyte counts in blood [3]. Notably, CD4+ T cells are critical for the development of allergic inflammatory diseases. CD4+ T cell activation induces the secretion of cytokines and chemokines and drives inflammation and allergic sensitization [4]. Furthermore, the development of AD has been attributed to the activation of mast cells [5, 6] and T-helper 2 (Th2)-dependent cells [7, 8]. Mast cells are activated by IgE through the high-affinity IgE receptor (Fc휀R) [9, 10]. These cells are then recruited into AD skin lesions, where they promote skin hypersensitivity reactions by releasing histamine; prostaglandin D2 (PGD2); AD-related Th2 cytokines, including IL-4, IL-5, and IL-13; and proinflammatory cytokines, including IL-4 and IL-6.

Tacrolimus is an effective immunosuppressant that inhibits the production of various cytokines, such as IL-2, IL-4 and IL-5. Many studies have demonstrated that tacrolimus suppresses allergic cytokine production by T cells [11, 12] and is effective against AD in animal models [13–15]. Tacrolimus ointment is used for the treatment of AD in adults and children [16–18]. However, previous studies have shown that treatment with tacrolimus elevated total and specific IgE levels and caused transient burning and erythema in ~ 60% of patients [19, 20]. Consequently, the development of alternative remedies is necessary to reduce these side effects.

Jawoongo is a traditional herbal medicine composed of Lithospermum root and Angelica gigas Nakai (AGN). AGN contains numerous active ingredients, including decursin. In previous studies, decursin exhibited anti-allergic effects in an asthma model and anti-metastatic effects in colon cancer [21–23]. Decursin has also been used for the treatment of various dermatitis-associated skin diseases, including eczema and chilblain. Recent studies have indicated that decursin is effective in driving artificial wound healing and ameliorating skin inflammation [24–26]. As known as, DNCB allergens elicited a systemic immune response, because increased cytokine levels in the serum of mice [27].

We investigated effect of jawoongo in DNCB induced AD model in Balb/c mice. The goal of this study was to explore the effects of Jawoongo on 2,4-dinitrochlorobenzene (DNCB)-induced AD-like symptoms in BALB/c mice and several types of immune cell.

## Methods

### Preparation of Jawoongo ointment and tacrolimus ointment

Jawoongo ointment was supplied by Han-poong Pharm Co., Ltd. (Jeonjoo, Republic of Korea). Jawoongo is made from Lithospermum root and *Angelica gigas Nakai* (AGN). The main compound in Lithospermum root is shikonin. Shikonin inhibits inflammation and imflammasomes [28, 29]. The main compounds in AGN are decursin and Nodakenin. Decursin and Nodakenin are known to inhibit inflammation. Additionally, decursin is known to inhibit the proliferation of ovarian cancer cells [30–32]. A 0.1% protopic tacrolimus ointment was also utilized (Astellas Pharma Tech, Japan). Tacrolimus is made from FK506, which has been used to treat dermatitis, as it suppresses the development of Th2 cells [33, 34].

### Animals

Six-week-old male BALB/c mice were obtained from Orient Bio, Inc. (Seoul, Korea). The mice were maintained for 1 week under a controlled temperature (23 ± 3 °C) and humidity (55 ± 15%) with a 12 h light/12 h dark cycle before initiating the experiment. The body weights and food intake of the animals were measured once every 2 days. All procedures performed on the mice were approved by the animal care center of Kyung-Hee University (Kyung Hee University Study Proposal (SEOUL) – 12-014; Approval No. KHUASP(SE)-12–014). Upon completion of the experiment, the mice were anesthetized with a 1.2% avertin solution (0.5 g 2,2,2-tribromoethanol powder dissolved into 1 ml 2-methyl-2-butanol and 39 ml phosphate-buffered saline (PBS) at 55 °C) that was filtered through a Nalgene 0.22-μm filter (Thermo Fisher Scientific, Inc., Waltham, MA, USA) and sacrificed via exsanguination [35, 36].

### Induction of AD-like lesions and drug treatment

**Fig. 1:**
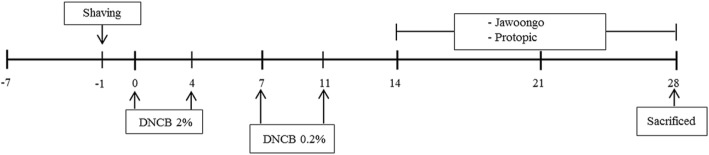
General schematic diagram for the study protocol

The procedures used for the induction of AD-like lesions and the drug treatment are shown in Fig. 1. The mice were divided into four groups, with eight mice in each group: group 1, normal; group 2, DNCB; group 3, DNCB + tacrolimus; group 4, DNCB + Jawoongo. After shaving, the back skin of the mice was painted with 200 μL of a 2% DNCB solution over 1 × 1 cm patches for one week and challenged again with 200 μL of a 0.2% DNCB solution twice a week. Tacrolimus or Jawoongo was then applied to the sensitized skin for two weeks. Following the last application of Tacrolimus or Jawoongo, the mice were sacrificed to perform immunological and histological assessments. The method was performed as described previously [37].

### Histological analysis

Skin samples (20 μm thick) were embedded in Tissue-Tek optical cutting temperature (OCT) compound (Leica, CA, Richmond, USA). The skin samples were stained with hematoxylin and eosin (H&E) to visualize inflammatory cells and with toluidine blue (TB) to visualize mast cells and then examined under a light microscope (Olympus). The mast cells and inflammatory cells were counted in 10 sections of high-power fields (HPFs) at 40×, 400× and 1000× magnification.

### Immunohistochemistry [38]

CD4+ lymphocytes were detected by immunohistochemical analysis using anti-CD4+ antibodies (Santa cruz biotechnology, Dallas, Texas, USA). After deparaffinization, the slides were rehydrated and antigen retrieval done by microwave treatment, they were treated with 3% hydrogen peroxide in PBS for 15 min to inhibit the endogenous peroxidase activity of blood cells. Following the hydrogen peroxide treatment, the sections were incubated with 5% bovine serum albumin (BSA) in PBS as a blocking reagent for 1 h at room temperature. The sections were then incubated with mouse monoclonal CD4+ antibodies (1:100 dilution) overnight at 4 °C. After washing with PBS, subsequently incubated with secondary biotinylated anti-rabbit IgG for 1 h at room temperature. The sections were treated with an avidin-biotin HRP complex (Vectastain ABC kit, Vector Labs, CA, USA) for 30 min at 4 °C and stained with diaminobenzidine (DAB) tetrachloride as a substrate. The slides were mounted in an aqueous mounting solution (DAKO, Glostrup, Denmark) and cover-slipped. All of the sections were analyzed using an Olympus microscope, and images were captured using a digital video camera.

### Analysis of mouse blood

Whole blood samples were collected by cardiac puncture and placed in Vacutainer TM tubes containing EDTA (BD Biosciences, USA) to prevent clotting. Anti-coagulated blood was processed to determine leukocyte counts, including lymphocytes, monocytes, eosinophils, basophils and neutrophils, using a HEMAVET 950 hematological analyzer (Drew Scientific, Inc., Oxford, USA).

### RT-PCR

RNA was isolated using an Easy-blue RNA Extraction Kit (iNtRON biotech, Republic of Korea). In brief, we harvested cells (HMC-1, RAW264.7 and Splenocyte cells) and mouse tissue and 1 ml of R&A-BLUE solution was added to each. Following this, 200 μl of chloroform was added to the lysate and then vigorously vortexed for 15 s. Then, the lysate was centrifuged at 13,000 rpm for 10 min at 4 °C. We then transferred the appropriate volume of the aqueous phase into a clean tube, added 400 μl of isopropanol and mixed the solution thoroughly by inverting the tube 6–7 times. After centrifuging the tube at 13,000 rpm for 10 min, the supernatant was carefully removed without disturbing the pellet. Then, 1 ml of 75% ethanol was added, and the solution was thoroughly mixed by inverting the tube 4–5 times. The mixture was then centrifuged for 1 min at room temperature, and the supernatant was carefully discarded without disturbing the pellet. Finally, the remaining RNA pellet was dried and then dissolved in 20–50 μl of RNase-free water. The concentration of the isolated RNA was determined using a NanoDrop ND-1000 spectrophotometer (NanoDrop Technologies Inc., Wilmington, USA). We treated DNase to each sample. Two micrograms of total cellular RNA from each sample was reverse-transcribed using a cDNA synthesis kit (TaKaRa, Otsu, Shinga, Japan). PCR was conducted in a 20 μL reaction mixture consisting of a DNA template, 10 pM of each gene-specific primer, 10× Taq buffer, 2.5 mM dNTP mixture, and 1 unit of Taq DNA polymerase (Takara, Otsu, Shinga, Japan). PCR was performed using the specific primers listed in Table 1.

**Table 1:** PCR primer sequences

Primer Type	Primer name	Primer Sequence
Mouse	IL-2	F: 5’-GCA GCT GTT GAT GGA CCT AC-3′
		R: 5’-TCC ACC ACA GTT GCT GAC TC-3′
	IL-4	F: 5’-TCG GCA TTT TGA ACG AGG TC-3′
		R: 5′-GAA AAG CCC GAA AGA GTC TC-3′
	IL-13	F: 5’-CGG CAG CAT GGT ATG GAG TG-3′
		R: 5′-ATT GCA ATT GGA GAT GTT GGT CAG-3′
	iNOS	F: 5′-AAT GGC AAC ATC AGG TCG GCC ATC ACT-3′
		R: 5’-GCT GTG TGT CAC AGA AGT CTC GAA CTC-3′
	TNF-α	F: 5′-ATG AGC ACA GAA AGC ATG ATC-3′
		R: 5’-TAC AGG CTT GTC ACT GGA ATT-3’
	GAPDH	F: 5′-GAG GGG CCA TCC ACA GTC TTC-3’
		R: 5’-CAT CAC CAT CTT CCA GGA GCG-3’
Human	IL-4	F: 5’-TGC CTC CAA GAA CAC AAC TG-3’
		R: 5’-CTC TGG TTG GCT TCC TTC AC-3’
	IL-6	F: 5’-AAC CTT CCA AAG ATG GCT GAA-3’
		R: 5’-CAG GAA CTG GAT CAG GAC TTT-3’
	IL-13	F: 5′-GGT CAA CAT CAC CCA GAA CC-3’
		R: 5′-TTT ACA AAC TGG GCC ACC TC-3’
	TSLP	F: 5′-TAT GAG TGG GAC CAA AAG TAC CG-3’
		R: 5′-GGG ATT GAA GGT TAG GCT CTG G-3’
	GAPDH	F: 5’-CGT CTT CAC CAC CAT GGA GA-3’
		R: 5’-CGG CCA TCA CGC CAC AGT TT-3’

### Enzyme-linked immunosorbent assay

Levels of IgE, IL-4, IL-6, IL-10, IL-12 and IL-13 were assessed using a Duoset enzyme-linked immunosorbent assay (ELISA) system (BD Biosciences, USA) according to the manufacturer’s instructions. In brief, to assess the level of IgE, IL-4, IL-6, IL-10, IL-12 and IL-13 in the mice serum treated with Tacrolimus and Jawoongo, 96-well plates were coated with capture antibody in ELISA coating buffer and incubated overnight at 4 °C. The plates were then washed with PBS with 0.05% Tween 20 (PBS-T) and subsequently blocked with 10% FBS in PBS for 1 h at 20 °C. Serial dilutions of standard antigen or sample in dilution buffer (10% FBS in PBS) were added to the plates, and the plates were incubated for 2 h at 20 °C. After the plates were washed, biotin-conjugated anti-mouse IgE and streptavidin-conjugated horseradish peroxidase (SAv-HRP) were added to the plates, and the plates were incubated for 1 h at 20 °C. Finally, the tetramethylbenzidine (TMB) substrate was added to the plates, and after 15 min of incubation in the dark, 2 N H_2_SO_4_ was added to stop the reaction. The optical density was measured at 450 nm on an automated ELISA reader. (Versa Max, Molecular Devices, CA, USA).

### Detection of nitric oxide

Nitric oxide (NO) production from RAW264.7 cells in culture was measured using Griess reagent (Welgene, Korea). Briefly, 150 μL of cell culture supernatant was mixed with 150 μL of Griess solution and incubated for 30 min at room temperature. The optical density was determined at 570 nm using a microplate reader.

### Cell viability assay

An MTS assay was performed to determine cell viability. To accomplish this, cells (HMC-1, RAW264.7 and Splenocyte cells) were seeded into a 96-well plate at a density of 3 × 10^3^ cells per well and treated 24 h later with varying concentrations of Jawoongo (5–500 μg/mL) for an additional 24 h. Ten microliters of WST solution was added to each well of the plate, which was incubated in the dark at 37 °C for another 2 h. Optical density was measured at 450 nm using an ELISA plate reader.

### Western blot analysis

Cells (HMC-1, RAW264.7 and Splenocyte cells) were lysed with cell lysis buffer (50 mM Tris-Cl pH 7.4, 1% NP-40, 0.25% sodium deoxycholate, 0.1% SDS, 150 mM NaCl, 1 mM EDTA, and protease inhibitor). Twenty micrograms of protein was separated by SDS-polyacrylamide gel electrophoresis and transferred to a nitrocellulose membrane (Protran nitrocellulose membrane, Whatman, UK). The membrane was blocked with 5% nonfat milk, probed with specific primary antibodies, incubated with HRP-conjugated secondary IgG antibodies (Calbiochem, San Diego, CA, USA), and visualized using an enhanced chemiluminescence detection system (Amersham ECL kit, Amersham Pharmacia Biotech Inc., Piscataway, NJ, USA). The antibodies against COX-2, p-JNK, total JNK and iNos were obtained from Cell Signaling (Danvers, MA, USA). The antibodies against p-Erk, total Erk, phospho-NF-κB p65 (Ser536), total NF-κB and Actin were obtained from Santa Cruz Biotechnology (Dallas, Texas, USA). Tubulin antibody was obtained from Sigma-Aldrich (St. Louis, MO, USA).

### Isolation of Splenocytes

Spleen suspensions from normal mice were prepared under aseptic conditions by homogenization in RPMI-1640 medium (containing 10% FBS, 1% antibiotics, and 0.05 mM β-mercaptoethanol). Red blood cell (RBC) lysis buffer (Sigma, St. Louis, MO, USA) was added to the cell suspension to remove RBCs. The spleen cells were centrifuged, suspended in complete RPMI-1640, and maintained at 37 °C in a humidified incubator with 5% CO_2_.

### Liquid chromatography-mass spectrometry analysis.

An Agilent 1100 series liquid chromatography-mass spectroscopy (LC-MS) with an atmospheric pressure chemical ionization interface was used in negative and positive ionization modes. Data were collected using Chemstation software version A.09.03. A Shiseido capcell-pak C18 column (4.6 mm × 150 mm, 5 μm) was used with an injection volume of 10 μL for the HPLC separation. The mobile phases consisted of (A) Acetonitrile, (B) 0.1% Acetic acid and (C) Methanol at a flow rate of 1.0 mL/min. The gradient of the mobile phases (A: B: C) for separation was 0–90 min (35: 65: 0 to 0: 0: 100). Decursin was used as standard. Mass spectrometry was operated with an electrospray ionization source and positive mode.

### Statistical analysis

All experiment results were expressed as the means ± SEM of at least three separate tests. Statistical significance at *P* < 0.05 < 0.01 and < 0.001 has been given respective symbols in the figures. Statistical analyses (ANOVA) were performed using PRISM software (GraphPad Software Inc., La Jolla, CA, USA,).

## Results

### Effects of Jawoongo on a DNCB-induced mouse model of AD

**Fig. 2:**
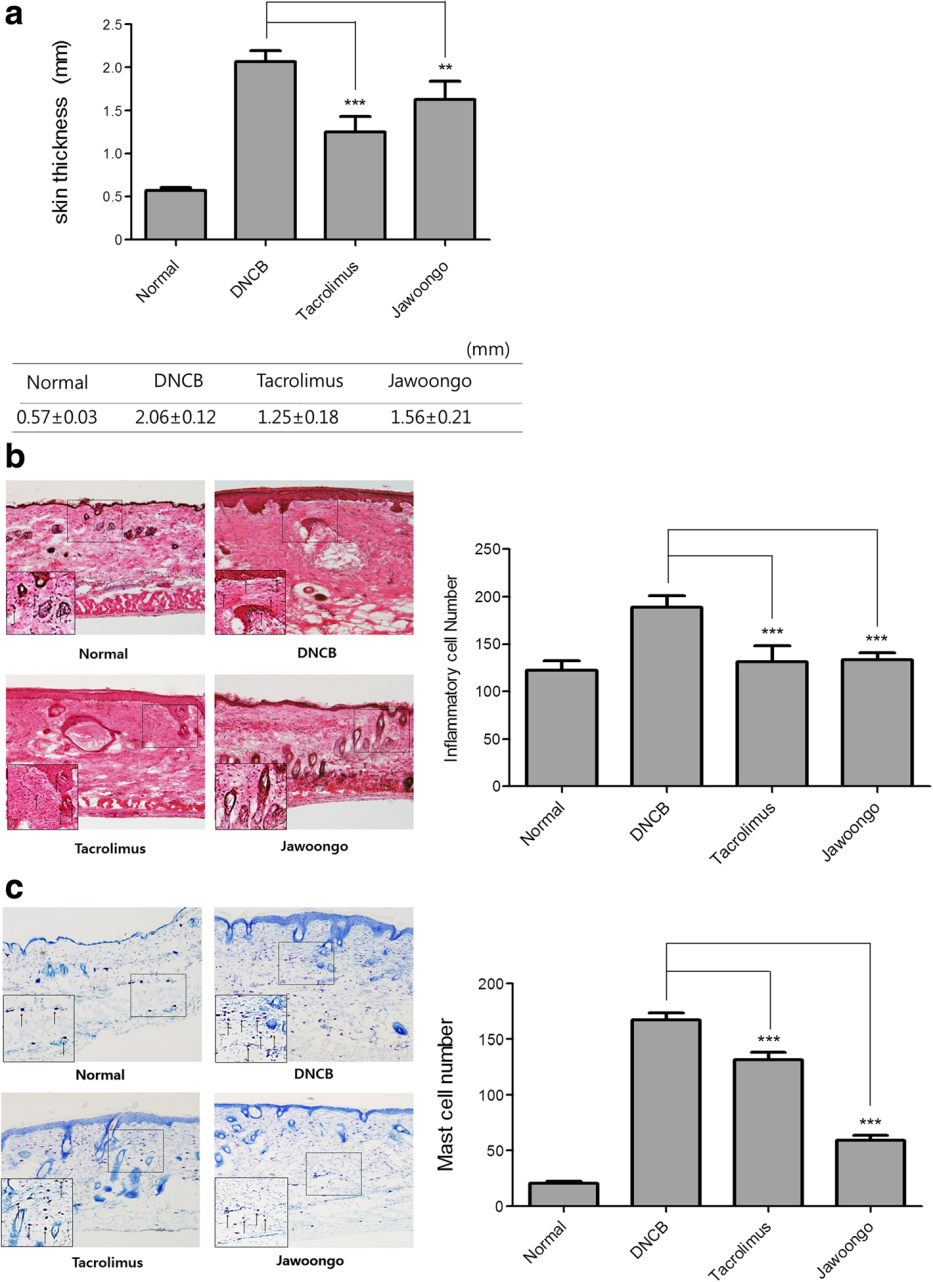
Effects of Jawoongo on the skin of mice with DNCB-induced AD. Skin thickness in mice with DNCB-induced AD that were treated with Jawoongo (**a**). Jawoongo reduced the infiltration of inflammatory cells into the skin. Skin sections were stained with hematoxylin and eosin (**b**). Arrows indicate inflammatory cells. Jawoongo reduced the infiltration of mast cells into the skin. Skin sections were stained with toluidine blue (**c**). Arrows indicate inflammatory cells. The sections were evaluated under a microscope at an original magnification of 200×. The data were presented as mean ± SEMs (*n* = 8 mice/group). *P < 0.05, **P < 0.01 and ***P < 0.001 as compared to DNCB-stimulated group, respectively

**Fig. 3:**
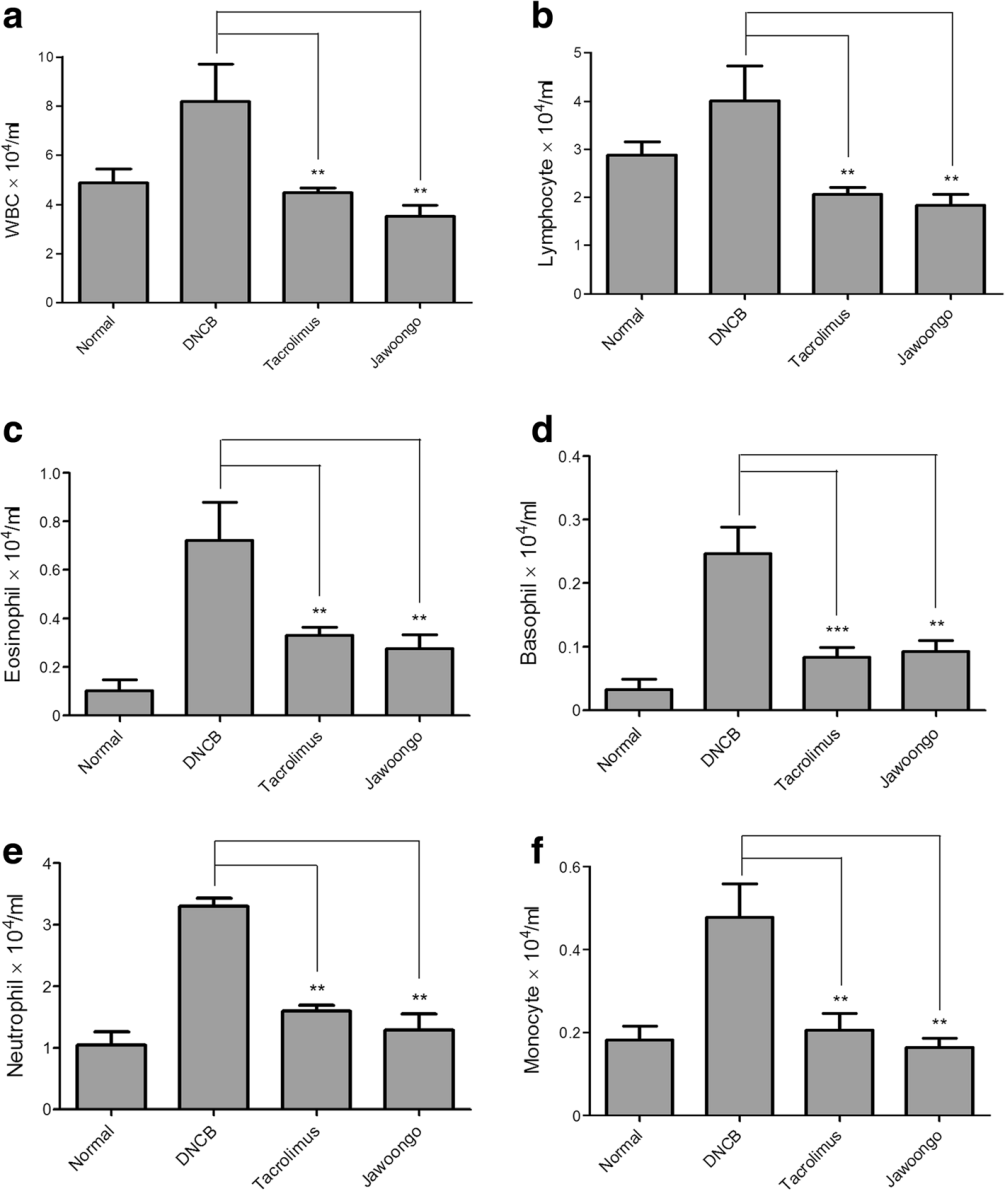
Jawoongo reduced leukocyte numbers in the blood. Blood samples were analyzed using a HEMAVET 950 hematological analyzer. The data were presented as mean ± SEMs (*n* = 8 mice/group). *P < 0.05, **P < 0.01 and ***P < 0.001 as compared to DNCB-stimulated group, respectively

We investigated the effects of Jawoongo on DNCB-induced AD-like symptoms. We found that skin thickness increased following application of DNCB (2.06 ± 0.12 mm) compared to no treatment (0.57 ± 0.03 mm), and this increase was inhibited by both Jawoongo (1.56 ± 0.21 mm) and tacrolimus (1.25 ± 0.18 mm) treatment (Fig. 2a). In addition, we monitored the body weights and food intake of the mice throughout the study and observed no significant changes, suggesting that Jawoongo did not produce any toxic effects on the mice (Additional file 1: Figure S1A and B). H & E staining was performed to examine whether Jawoongo reduces the infiltration of inflammatory cells into the skin. The number of inflammatory cells in the mice with DNCB-induced AD lesions was higher than that in the normal mice and decreased following treatment with Jawoongo or tacrolimus (Fig. 2b, left panel). The bar graph indicates the average number of cells counted from a random field of view (Fig. 3b, right panel). Moreover, toluidine blue staining was used to examine whether Jawoongo reduces mast cell infiltration into the skin. There was greater mast cell infiltration in the skin of the mice with DNCB-induced AD lesions than that of the normal mice. Treatment with Jawoongo or tacrolimus decreased the infiltration of mast cells into the skin (Fig. 2c, left panel). The bar graph indicates the average number of cells counted from a random field of view (Fig. 2c, right panel).

### Jawoongo treatment lowered the number of WBCs in the blood of mice

Application of DNCB increased both the total number of white blood cells (WBCs) and the number of each WBC subtype, including neutrophils, basophils, eosinophils, monocytes, and lymphocytes, in the serum of the mice. Importantly, treatment with Jawoongo or tacrolimus lowered the increased number of WBCs, indicating that Jawoongo and tacrolimus suppress inflammation by decreasing the number of WBCs in the blood (Fig. 3a, b, c, d, e and f).

### Jawoongo treatment reduced the serum levels of IgE, IL-6, IL-10 and IL-12 in mice

**Fig. 4:**
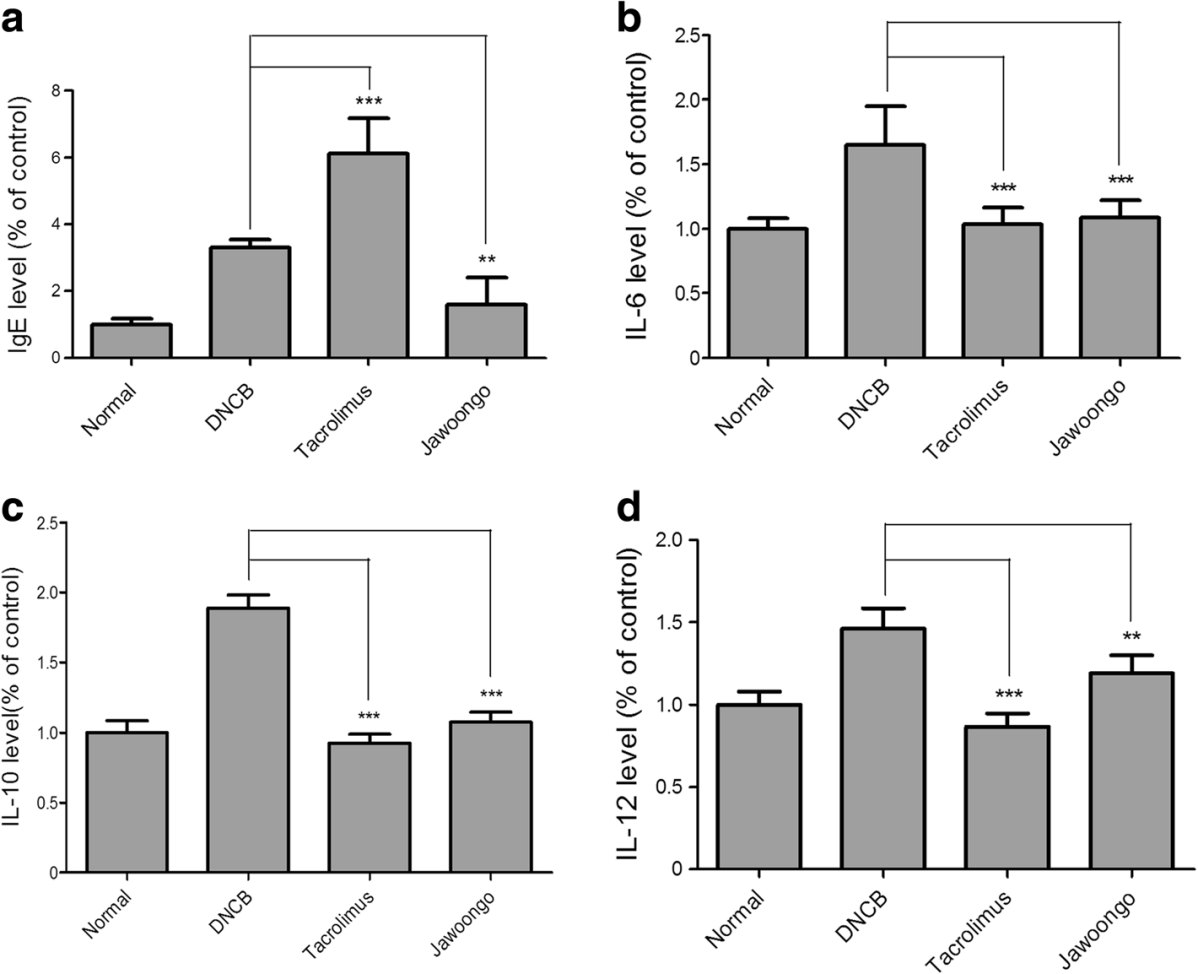
Jawoongo reduced cytokine levels in serum. Cytokine levels were measured by ELISA. The data were presented as mean ± SEMs (*n* = 8 mice/group). *P < 0.05, **P < 0.01 and ***P < 0.001 as compared to DNCB-stimulated group, respectively

We next measured proinflammatory cytokine levels by ELISA. We found that Jawoongo treatment reduced the serum levels of IgE, IL-6, IL-10 and IL-12 whose expression was induced after DNCB application. Tacrolimus treatment increased the serum levels of IgE (Fig. 4a, b, c and d).

### Jawoongo treatment down-regulated mRNA expression of IL-2, IL-4, IL-13, and TNF-α in mouse skin

**Fig. 5:**
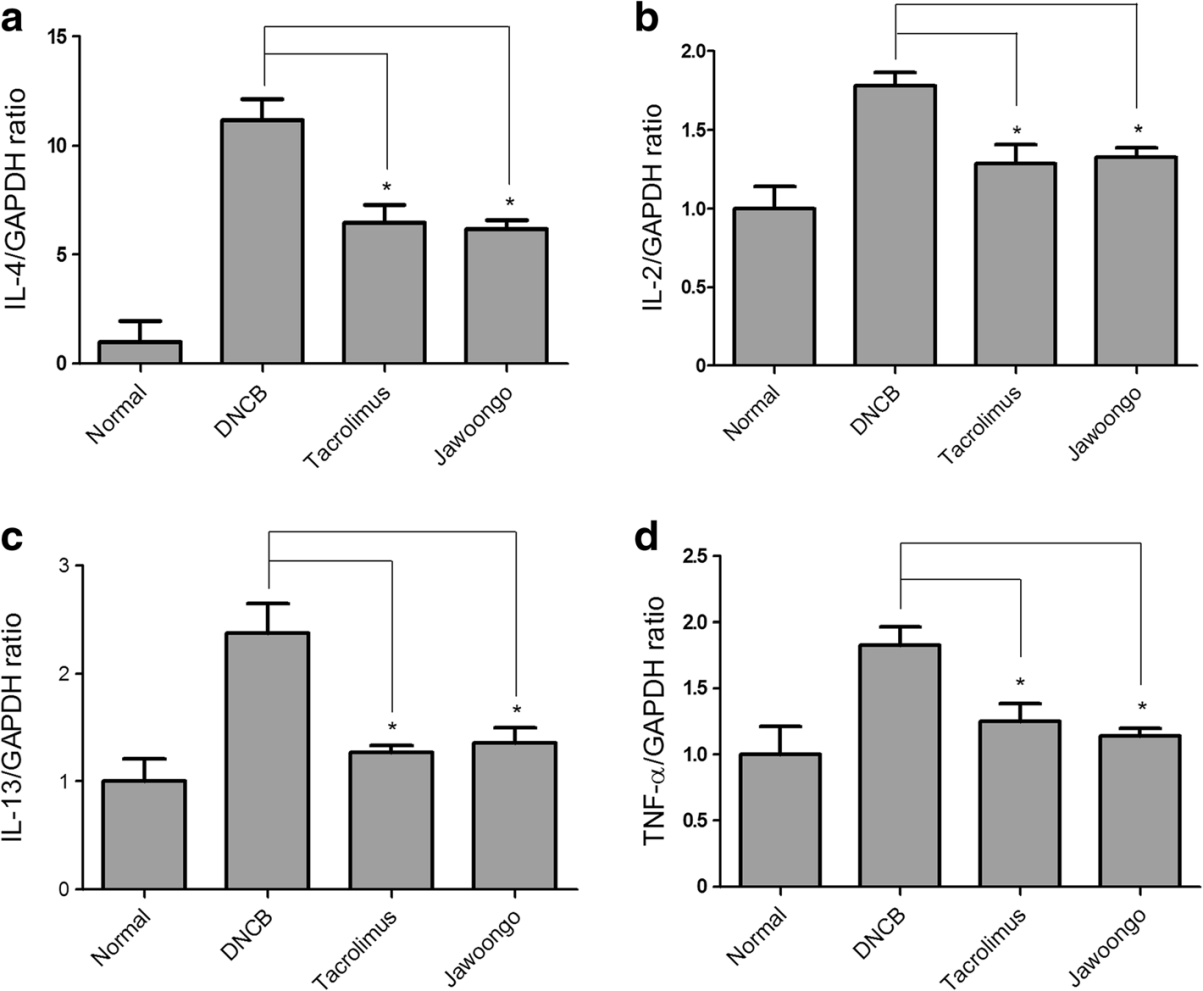
Effects of Jawoongo on cytokine mRNA expression in mouse skin tissue. IL-2, IL-4, IL-13 and TNF-α mRNA expression was measured by RT-PCR, shown in (**a**), (**b**), (**c**), and (**d**), respectively, in mouse skin tissue. The data were presented as mean ± SEMs (*n* = 8 mice/group). *P < 0.05, **P < 0.01 and ***P < 0.001 as compared to DNCB-stimulated group, respectively

RT-PCR analysis of RNA extracted from mouse skin revealed that DNCB increased the levels of AD-associated cytokines such as IL-2, IL-4, IL-13 and TNF-α when applied to skin, and subsequent treatment with Jawoongo or tacrolimus suppressed the increased cytokine levels (Fig. 5a, b, c and d).

### Jawoongo treatment reduced the number of CD4+ cells in skin

**Fig. 6:**
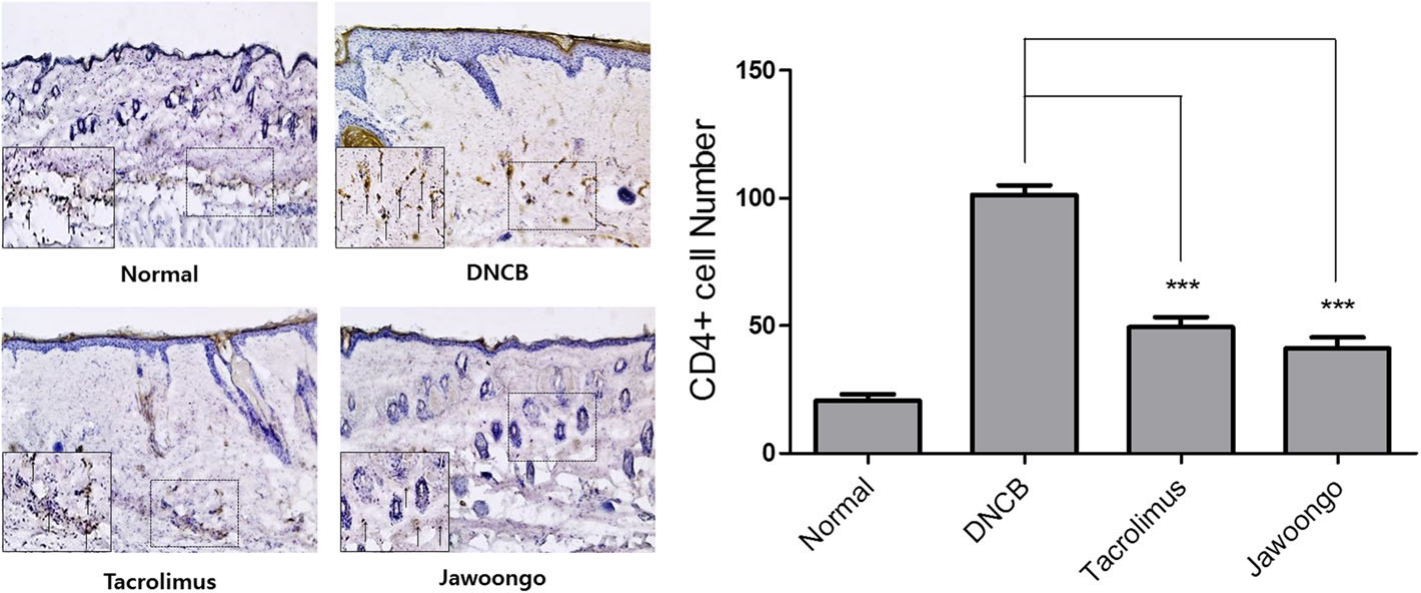
Distribution of CD4+ cells in the skin of mice with DNCB-induced AD. Skin sections were immunostained with CD4+ antibodies. CD4+ cells display a brown color. The sections were evaluated under a microscope at an original magnification of 200×

We performed immunocytochemistry to examine whether Jawoongo treatment can reduce the level of CD4+ in skin. The level of CD4+ in the mice with DNCB-induced AD lesions was higher than that in the normal mice. Additional treatment with Jawoongo or tacrolimus decreased the number of CD4+ cells (Fig. 6).

### Jawoongo inhibited agonist-induced cytokine production in HMC-1 cells

**Fig. 7:**
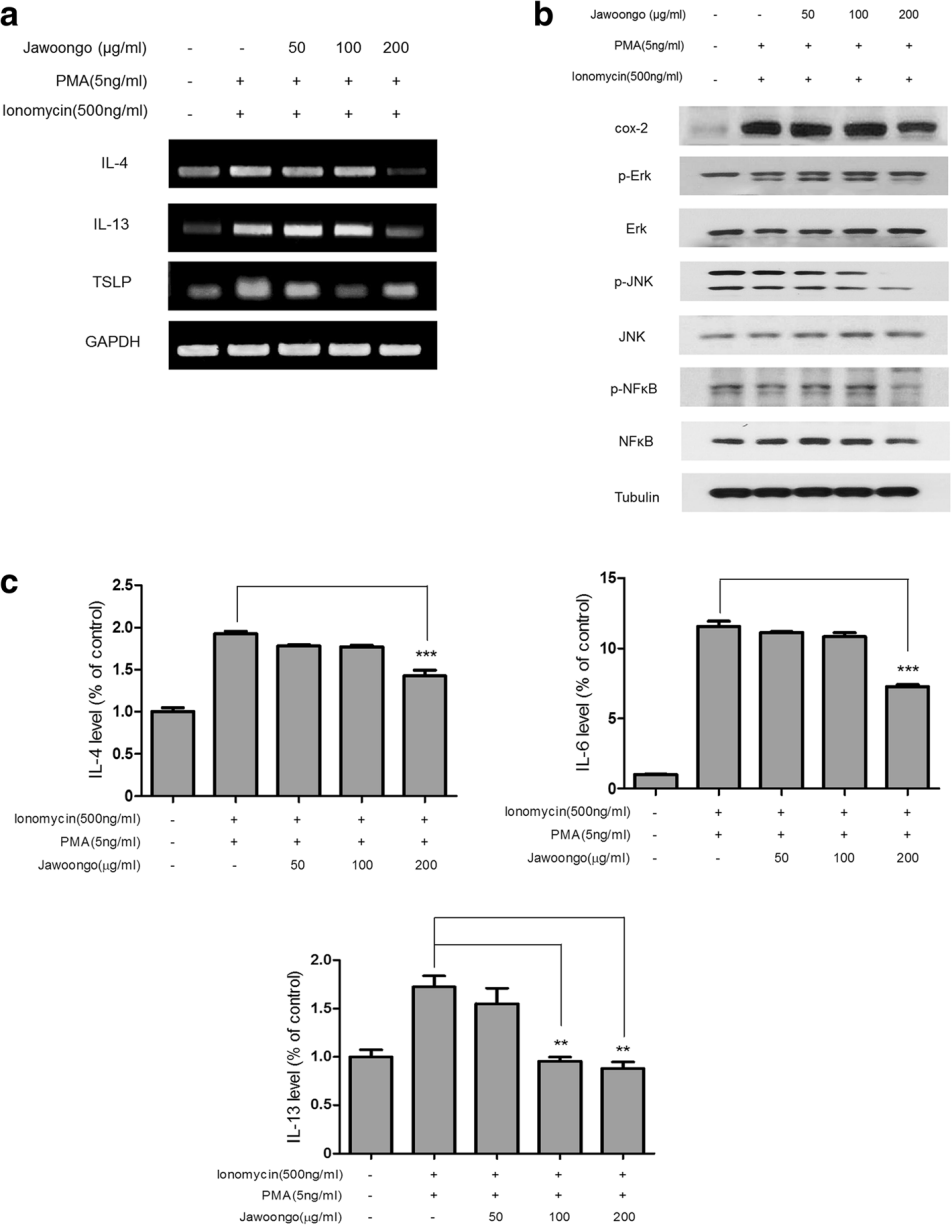
Effects of Jawoongo on cytokine expression in HMC-1 cells. HMC-1 cells were stimulated with ionomycin (500 ng/ml) and PMA (5 ng/ml) and then treated with different concentrations of Jawoongo (50–200 μg/ml) for 24 h. IL-4, IL-13 and TSLP mRNA expression was measured by RT-PCR (**a**). Whole cell lysates were analyzed by Western blotting (**b**). The culture medium of the cells was harvested, and IL-4, IL-6 and IL-13 cytokine levels were measured by ELISA (**c**). The data were presented as mean ± SEMs (*n* = 8 mice/group). *P < 0.05, **P < 0.01 and ***P < 0.001 as compared to Ionomycin and PMA-stimulated group, respectively

Based on the observation that Jawoongo inhibited AD-associated cytokine production in mice, we next investigated whether Jawoongo affects cytokine expression in human mast cell line 1 (HMC-1) cells. To accomplish this, HMC-1 cells were stimulated with ionomycin and PMA before treatment with varying concentrations of Jawoongo. RT-PCR analysis showed that Jawoongo dose-dependently suppressed the IL-4, IL-13 and TSLP mRNA expression that was induced by treatment with ionomycin and PMA (Fig. 7a). Moreover, Western blot analysis indicated that Jawoongo significantly reduced agonist-stimulated Erk, JNK, NF-κB and COX-2 protein expression in a dose-dependent manner (Fig. 7b). We also demonstrated that Jawoongo inhibited agonist-stimulated IL-4, IL-6, and IL-13 secretion, as determined by ELISA (Fig. 7c). No significant effect on cell viability was observed in HMC-1 cells treated with Jawoongo alone or in combination with ionomycin and PMA (Additional file 2: Figure S2A).

### Jawoongo inhibited LPS-induced inflammatory responses in RAW264.7 cells

**Fig. 8:**
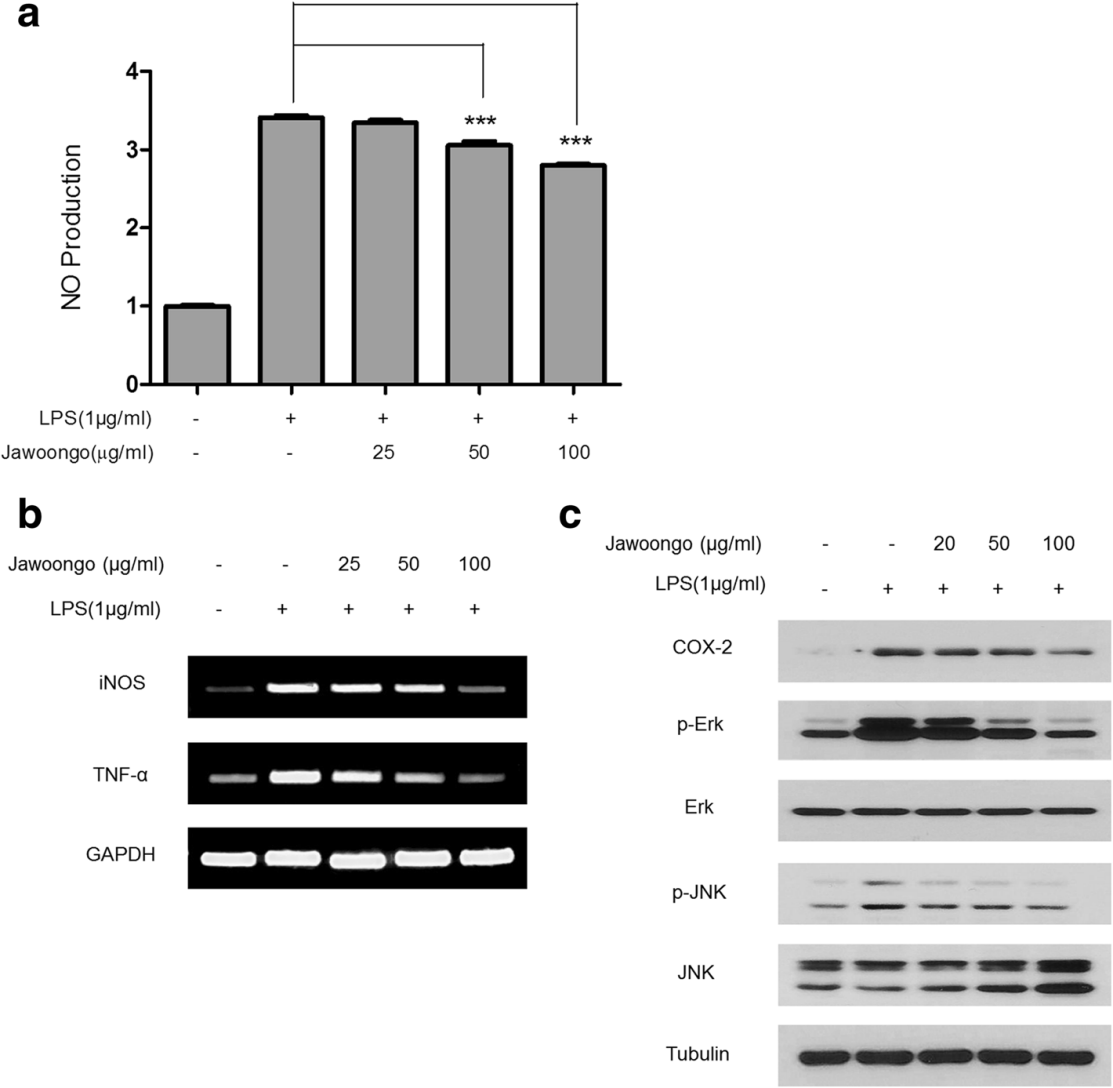
Effects of Jawoongo on NO production in RAW264.7 cells. RAW264.7 cells were stimulated with LPS (1 mg/ml) and then treated with different concentrations of Jawoongo (25–100 μg/ml) for 24 h. NO production was measured using the Griess reagent system (**a**). iNOS and TNF-α mRNA expression was measured by RT-PCR (**b**). Whole cell lysates were analyzed by Western blotting (**c**). The data were presented as mean ± SEMs (*n* = 8 mice/group). *P < 0.05, **P < 0.01 and ***P < 0.001 as compared to LPS-stimulated group, respectively

Because NO plays an important role in allergic responses, we next examined the effects of Jawoongo on NO production and inducible nitric oxide synthase (iNOS) mRNA expression in the murine RAW264.7 macrophage cell line. As shown in Fig. 8a and b, Jawoongo reduced LPS-induced NO production and iNOS mRNA expression in a dose-dependent manner. Jawoongo also decreased LPS-induced increases in mRNA and protein expression of inflammation-related genes, such as tumor necrosis factor-α (TNF-α) and COX-2. Moreover, Jawoongo had an inhibitory effect on ERK and JNK activation in relation to various inflammatory responses (Fig. 8c). When using a low concentration of Jawoongo (~ 100 g/ml), no significant effect on RAW264.7 cell viability was observed (Additional file 2: Figure S2B).

### Jawoongo inhibited LPS-induced inflammatory responses in isolated Splenocytes

**Fig. 9:**
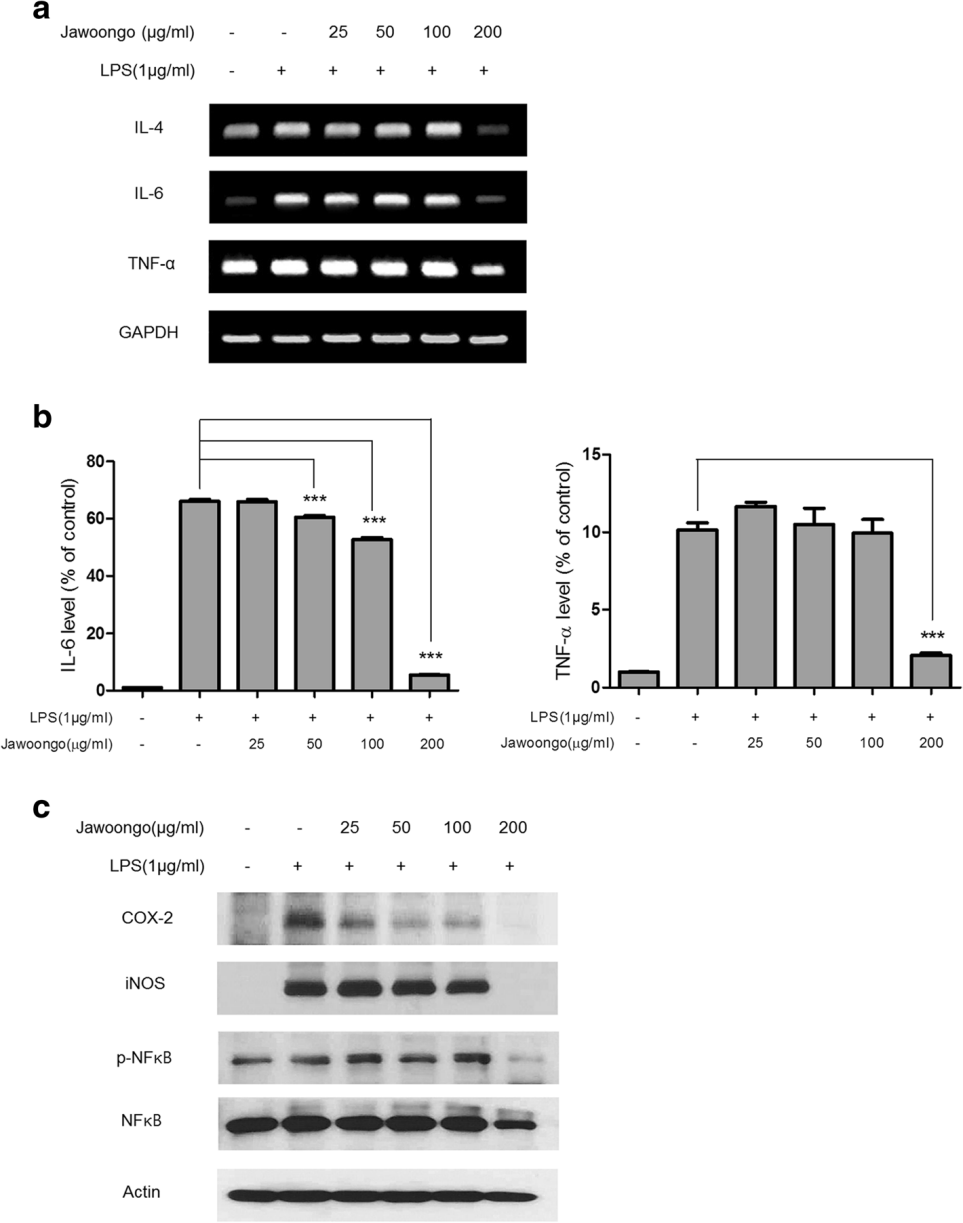
Effects of Jawoongo on cytokine expression in splenocytes. Splenocytes were stimulated with LPS (1 μg/ml) and then treated with different concentrations of Jawoongo (25–200 μg/ml) for 24 h. IL-4, IL-6 and TNF-α mRNA expression was measured by RT-PCR (**a**). The culture medium of the cells was harvested, and IL-6 and TNF-α cytokine levels were measured by ELISA (**b**). Whole cell lysates were analyzed by Western blotting (**c**). The data were presented as mean ± SEMs (*n* = 8 mice/group). *P < 0.05, **P < 0.01 and ***P < 0.001 as compared to LPS-stimulated group, respectively

We further tested Jawoongo’s anti-inflammatory activities in splenocytes isolated from mice. Similar to HMC-1 and RAW264.7 cells, Jawoongo treatment reduced the mRNA levels of proinflammatory cytokines, including IL-4, IL-6, and TNF-α in LPS-stimulated splenocytes (Fig. 9a). Dose-dependent inhibition of IL-6 and TNF-α secretion by Jawoongo was also observed (Fig. 9b). Finally, Western blot analysis demonstrated that treatment with a high dose of Jawoongo decreased COX-2 and iNOS levels and NF-KB activity in LPS-induced splenocytes (Fig. 9c). At a concentration of ~ 200 g/ml, as was used in these experiments, Jawoongo showed no significant toxic effects on splenocytes (Additional file 2: Figure S2C).

### Decursin is an Indicator molecule for Jawoongo

**Fig. 10:**
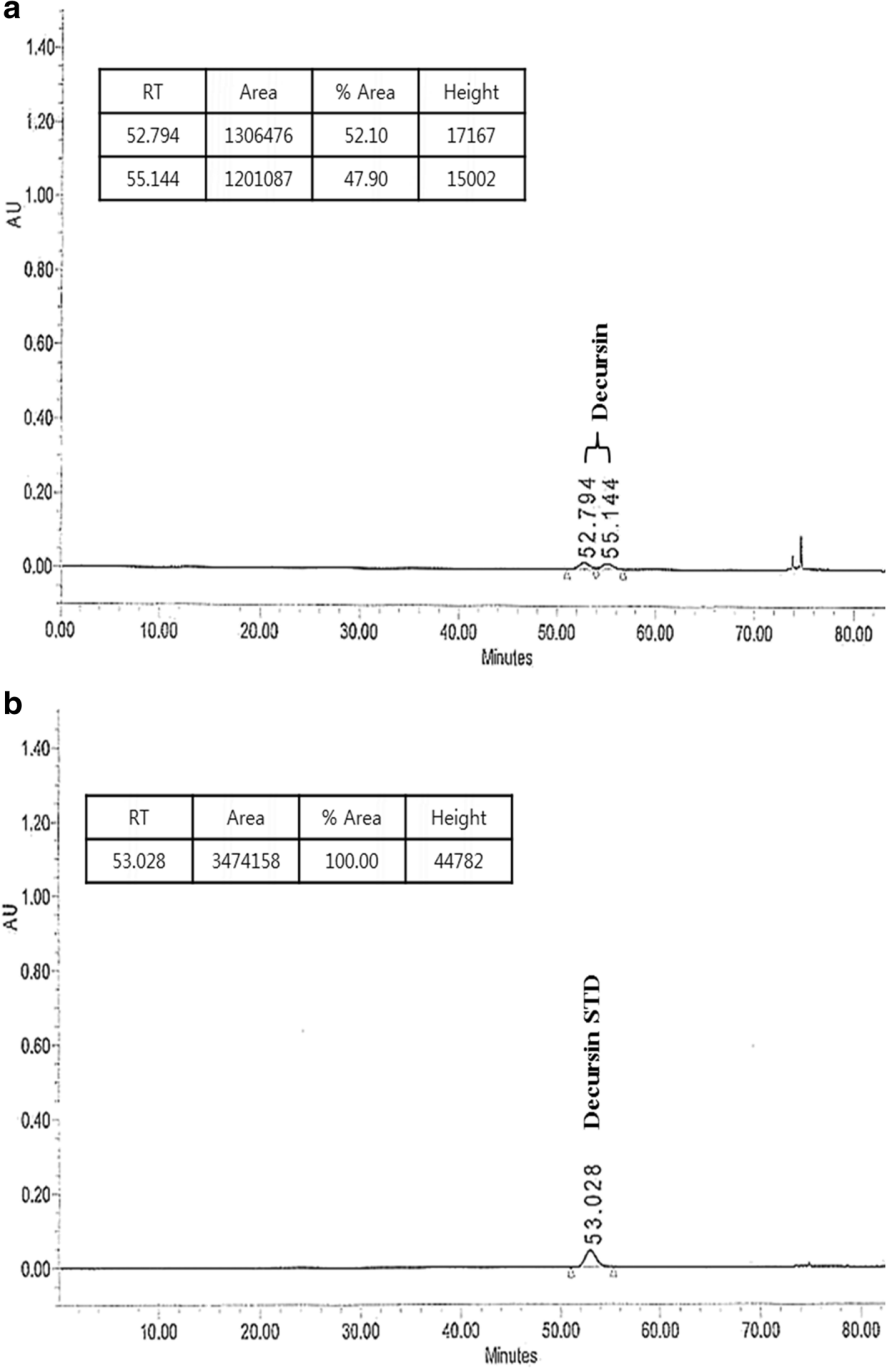
LC-MS chromatogram. **a** Identification of decursin in Jawoongo. **b** Mass spectrum peak at 53.028 min

Liquid chromatography-mass spectrometry was used to measure the retention time of decursin. Chromatograms were acquired at 215 nm on an HPLC by UV detection (Fig. 10a), and the retention time of decursin was 53.028 min (Fig. 10b).

## Discussion

AD is a common pruritic and chronically relapsing inflammatory skin disease. Affecting approximately 10–20% of children and 1–3% of adults worldwide [39], AD is a major global public health problem. Additionally, the incidence of AD has steadily increased every year [40].

Several mouse models have been developed to evaluate drugs for the treatment of AD. A DNCB-patch model using BALB/c mice has been proposed as a suitable representative of human AD because mice treated with DNCB show symptoms similar to human AD, including epidermal hyperplasia, dermal mast cell infiltration, and elevated serum IgE levels [41]. Activated mast cells release inflammatory mediators such as histamines, cytokines and chemokines [42, 43]. In this study, we investigated the anti-AD effects of Jawoongo using DNCB-treated BALB/c mice. We found that topical application of Jawoongo strongly suppressed DNCB-induced AD-like lesions and reduced skin thickness, CD4 levels and mast cell infiltration in sensitized skin. We observed that Jawoongo suppresses skin inflammation by inhibiting various DNCB-stimulated inflammatory responses.

Until now, the exact pathogenesis of AD has remained unclear. However, Th1 and Th2 cytokines play an important role in the etiology of AD. In particular, Th2 cytokines are important mediators of AD development [44]. CD4+ T cells are key factors implicated in the pathogenesis of AD, and skin infiltration of CD4+ T cells is known to increase in severe AD cases [45].

Therefore, we investigated cytokines related to Th2 in an in vivo model. Jawoongo treatment reduced the increased serum levels of IgE, IL-6, IL-10 and IL-12 induced by DNCB treatment In fact, it was reported that IL-12 was increased by DNCB [46, 47], and this increase was suppressed by Jawoongo as expected. We also found that Jawoongo reduced DNCB-stimulated increases in eosinophil, neutrophil, monocyte, basophil, lymphocyte and WBC numbers and in IL-4, IL-13 and TNF-α mRNA expression. These results suggest that Jawoongo decreased the number of CD4+ cells entering the skin. Of note, Jawoongo and tacrolimus, which was used as a positive control in this study, showed similar effects on AD-like skin lesions, but Jawoongo exhibited more favorable effects than tacrolimus in some aspects, such as in decreased mast cell recruitment and serum IgE levels.

To improve our understanding of Jawoongo’s actions at the cellular level, we evaluated the effects of Jawoongo on several types of innate immune cell, including human mast cells (HMC-1), murine macrophage RAW264.7 cells, and splenocytes isolated from mice.

**Fig. 11:**
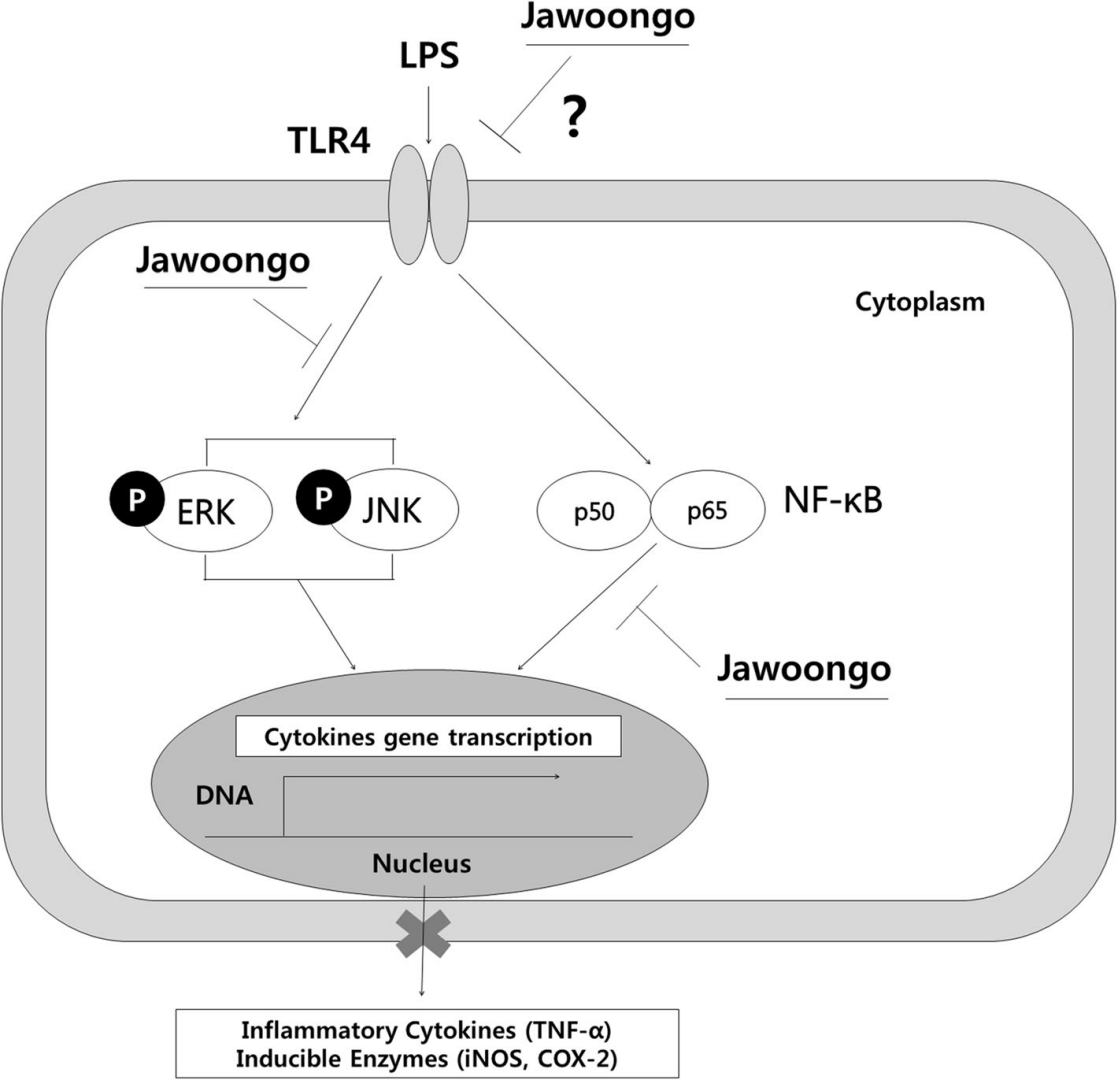
Pathway diagram of the mechanism of histamine inhibition of ERK, JNK and NF-kB activation. RAW264.7 cells and Splenocyte activated with a LPS agonist activated the MEK/ERK MAPK signaling and NF-kB signaling cascade to induce production of TNF. Jawoongo inhibited MEK/ERK and NF-kB activation and suppressed production of TNF

Inflammation causes cells to respond to stimulation by releasing various cytokines and by increasing COX-2 expression. Therefore, COX-2 expression can be measured to evaluate anti-inflammatory effects [48, 49]. In the present study, Jawoongo treatment suppressed AD-associated cytokine production, such as IL-4, Erk, JNK, p-NF-κB and COX-2 expression, in HMC-1 cells. iNOS produces NO after it is activated by various cytokines. Inflammatory and immune responses lead to vasodilation, erythema and edema in response to increasing NO levels. Excessive NO aggravates the inflammatory response due to the immune-regulatory role of NO [50, 51]. Macrophages contain several factors that regulate cytokine and chemokine secretion in AD. Therefore, macrophages have an important role in both the acute and chronic inflammation associated with AD [52–54]. Macrophages are involved in the initiation and maintenance of acute and chronic inflammatory responses [55]. Treatment of murine macrophage RAW264.7 cells with Jawoongo suppressed LPS-stimulated NO production, reduced iNOS and TNF-α mRNA levels, and decreased ERK and JNK activation. We also found that Jawoongo treatment reduced IL-4, IL-6 and TNF-α mRNA levels; COX-2 and iNOS protein levels; and NFκB activity in LPS-induced splenocytes. It appears that high concentrations of Jawoongo are required to mediate an effect in vitro. Because both MAP kinase and NFκB pathways are implicated in AD, Jawoongo appears to inhibit both pathways (Fig. 11). Taken together, our results suggest that Jawoongo regulates proinflammatory cytokine production in several types of immune cell, thereby suppressing the AD-like symptoms caused by DNCB.

## Conclusions

Our present study demonstrates that Jawoongo treatment suppresses DNCB-induced AD symptoms by downregulating serum IgE levels and the production of several inflammatory cytokines. In addition, our data indicate that Jawoongo treatment inhibits cytokine expression and activation of the NF-kB and MAPK pathways in several types of immune cell. Taken together, our results suggest that Jawoongo might be a useful candidate drug for the treatment of AD.

## Additional files

Additional file 1: **Figure S1.** Changes in body weight (A) and food intake (B) in DNCB-induced AD mice during treatment with Jawoongo. Values are expressed as the mean ± SEMs (*n* = 8). (TIF 4580 kb)

Additional file 2: **Figure S2.** Effects of Jawoongo on cell viability in various cell lines. HMC-1 cells were treated with the combination of ionomycin (500 ng/ml) and PMA (5 ng/ml) with varying concentrations of DMSO and Jawoongo (5–500 μg/ml) for 24 h (A). RAW264.7 cells (B) and splenocytes (C) were treated with the combination of LPS (1 mg/ml) and varying concentrations of DMSO and Jawoongo (5–500 μg/ml) for 24 h. Cell viability was measured using an MTS assay. The data were presented as mean ± SEMs of three independent experiments. *P < 0.05, **P < 0.01 and ***P < 0.001. (TIF 18563 kb)

## Abbreviations

AD: Atopic dermatitis
AGN: Angelica gigas Nakai
BSA: Bovine serum albumin
DAB: Diaminobenzidine
DNCB: 2,4-dinitrochlorobenzene
HMC-1: Human mast cells
IgE: Immunoglobulin E
iNOS: Inducible nitric oxide synthase
MTS: 3-(4,5-dimethylthiazol-2-yl)-5-(3-carboxymethoxyphenyl)-2-(4-sulfophenyl)-2H-tetrazolium
NO: Nitric oxide
OCT: Optical cutting temperature
PGD2: Prostaglandin D2
PMA: Phorbol-12-Myristate-13-Acetate
TB: Toluidine blue
Th2: T-helper 2
TNF-α: Tumor necrosis factor-α
TSLP: Thymic stromal lymphopoietin
WBCs: White blood cells
WST: Tetrazolium salt
